# Antibiotic treatment and antimicrobial resistance in urinary tract infections among the older population in Denmark, 1999 to 2023

**DOI:** 10.2807/1560-7917.ES.2026.31.33.2500801

**Published:** 2026-08-20

**Authors:** Majda Attauabi, Diana Schultz Christensen, Laura Marie Christensen, Jonas Kähler, Ute Wolff Sönksen, Anne Byriel Walls

**Affiliations:** 1 Department of Bacteria, Parasites and Fungi, Statens Serum Institut, Copenhagen, Denmark; 2 Department of Drug Design and Pharmacology, University of Copenhagen, Copenhagen, Denmark; 3 Data Integration and Analysis, Statens Serum Institut, Copenhagen, Denmark; 4 Capital Region Hospital Pharmacy, Rigshospitalet, Copenhagen, Denmark

**Keywords:** Urinary tract infection, Antimicrobial resistance, Antimicrobial use, Antibiotic prescriptions, Antibiotic Stewardship, Elderly/older population, Long-Term Care Facility, Primary Healthcare

## Abstract

**BACKGROUND:**

Antimicrobial resistance is highly correlated with inappropriate antimicrobial use. Urinary tract infections (UTIs) are the main infections treated with antibiotics in the population ≥ 70 years.

**AIM:**

We aimed to investigate antibiotic prescribing patterns for UTIs treatment among older people in Denmark and resistance trends to assess impact of antibiotic stewardship interventions and quality improvement strategies.

**METHODS:**

This national register-based surveillance study included people ≥ 70 years in Danish primary healthcare and assessed consumption of pivmecillinam, trimethoprim, nitrofurantoin and sulfamethizole from 1999 to 2023 and resistance levels from 2018 to 2023. Consumption data were obtained from the Register of Medicinal Product Statistics and resistance data from the Danish Microbiology Database. A review of quality improvement strategies from 2005 to 2020 was conducted to provide context for the study.

**RESULTS:**

The highest level of antibiotic consumption for UTIs in older people was in 2014 (447 prescriptions/1,000 people ≥ 70 years) and gradually decreased by 28.4% in 2023. Resistance to pivmecillinam, trimethoprim, sulfamethizole and nitrofurantoin in *Escherichia coli* and *Klebsiella pneumoniae* decreased significantly from 2018 to 2023, alongside the decline in antibiotic consumption. Focus on antibiotic stewardship was intensified after 2013, with several quality improvement initiatives and updated treatment guidelines aiming at improving diagnosis and antibiotic treatment.

**CONCLUSION:**

Antibiotic consumption for UTIs in people ≥ 70 years decreased notably, accompanied by a decrease in resistance levels, reflecting a positive impact of antibiotic stewardship programmes to improve diagnosis and treatment. However, a growing older population could place a strain on healthcare resources in the future.

**Key public health message**

**What did you want to address in this study and why?**

We wished to investigate trends in antibiotic consumption (1999–2023) and antimicrobial resistance (AMR) (2018–2023) among people aged 70 years and above who are treated for urinary tract infections (UTIs) in Denmark. We also wanted to investigate how quality improvement strategies may have impacted the trends in antibiotic consumption and AMR.

**What have we learnt from this study?**

The results show a decrease in the number of antibiotic prescriptions for UTIs in older people since 2014, alongside a decrease in AMR in the two main pathogens causing UTIs, *Escherichia coli* and *Klebsiella* *pneumoniae*. This may reflect the impact of initiatives implemented to improve diagnosis and treatment of UTIs in Denmark.

**What are the implications of your findings for public health?**

The growing older population results in a higher number of individuals at risk of a UTI. This indicates that, due to the positive trends in AMR and antimicrobial use (AMU), AMR in UTIs may not pose an acute threat at present in Denmark, but healthcare resources could be strained in the near future if AMR and AMU are not continuously monitored and addressed.

## Introduction

The World Health Organization has identified antimicrobial resistance (AMR) as one of the greatest threats to public health [1]. Development of AMR is correlated with excessive antibiotic use [2]. Resistance can complicate the treatment of infectious diseases and, in the worst cases, cause severe illness and death [1]. Denmark maintains low levels of antibiotic consumption; however, specific populations may still receive unnecessary antibiotic treatment. One of the main indications for antibiotic treatment in Danish primary healthcare is urinary tract infections (UTIs) in older people (≥ 70 years) [3,4]. In Danish general practice, UTIs are treated with pivmecillinam as first-line treatment. Alternatively, and in cases of penicillin allergy, nitrofurantoin or trimethoprim are recommended. Sulfamethizole is also a treatment option [5,6].

*Escherichia coli* is the most common uropathogen followed by *Klebsiella pneumoniae* [7]. Urinary tract infections are characterised by clinical symptoms including dysuria and pollakiuria, as well as bacteriuria [8]. However, UTIs in older people are often more difficult to diagnose because of the absence of typical symptoms. Clinical suspicion should instead arise in the presence of atypical symptoms, such as new or worsened urinary tract symptoms, fever and altered general condition [9].

The presence of bacteriuria without symptoms of UTI, i.e. asymptomatic bacteriuria (ABU) [5], should generally not be treated with antibiotics [10]. The frequency of ABU increases with age and comorbidities, and is common in older people living in long-term care facilities (LTCFs). The high prevalence of ABU in older people is a known risk factor of inappropriate antibiotic prescribing [10,11].

In addition to a higher prevalence of ABU among older people, comorbidities and age-related physiological changes increase the risk of infection [12]. A common condition that can cause UTIs in older patients is urinary retention [13], which can also be drug-induced. These conditions lead to high antibiotic consumption and may promote selection of resistant bacterial strains.

Residence in LTCFs is associated with higher risk of UTIs due to hygiene challenges in institutionalised environments [14]. According to The Danish Integrated Antimicrobial Resistance Monitoring and Research Programme (DANMAP) older people living in LTCFs receive significantly more antibiotics than adults from the same age group living in their own homes. This difference is mainly attributable to UTIs [15].

Surveillance studies play a central role in monitoring AMR and shaping public health policies that aim to improve patient outcome and promote overall public health [16]. The objective of this surveillance study was to investigate the consumption patterns of antibiotics used to treat UTIs in the older population ≥ 70 years in Denmark. This study further examined the relationship between antibiotic consumption and AMR trends, and the potential impact of quality improvement strategies on this relationship. The ultimate goal was to provide evidence for antibiotic stewardship interventions and guide future research.

## Methods

### National register-based surveillance study of antibiotic consumption

#### Data collection, study design and population

This register-based surveillance study is based on aggregated DANMAP data on antibiotic prescriptions in Danish primary healthcare, extracted in February 2024. The data originate from the Danish Civil Registry and the Danish Register of Pharmaceutical Sales at the Danish Health Data Authority for the period 1999–2023 [3].

We included all prescriptions for antibiotics for systemic use (Anatomical Therapeutic Chemical (ATC) code: J01) in Denmark during the study period for persons aged 70 years or older and antibiotics with an ATC code indicative of UTI treatment (Table 1).

**Table 1:** Antibiotics approved for treatment of urinary tract infections in Denmark

Anatomical therapeutic chemical code	Active substance	Status
J01CA08	Pivmecillinam	Included
J01EB02	Sulfamethizole	Included
J01EA01	Trimethoprim	Included
J01XE01	Nitrofurantoin	Included
J01MA02	Ciprofloxacin	Excluded

**Figure 1:**
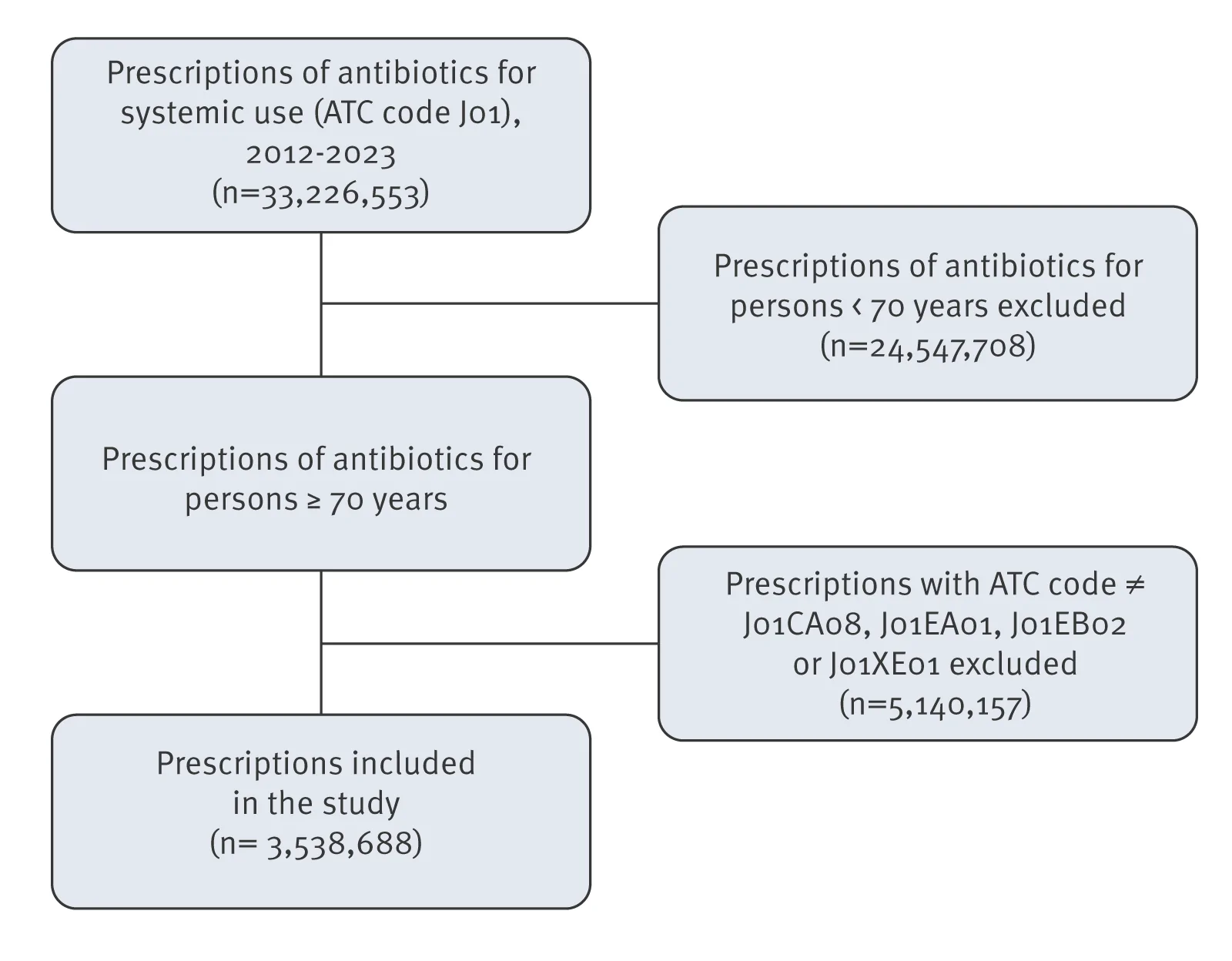
Flowchart of data filtering and selection for antibiotic prescription data, Denmark, 1999–2023

Among antibiotics, ciprofloxacin (ATC code: J01MA02), which is also approved in Denmark to treat UTIs, was excluded due to its approval for the treatment of several other infections [15]. The study population was further divided into two subgroups: 70–79 years and 80 years or older. Figure 1 provides an overview of the filtering process for prescription data.

ATC code: Anatomical Therapeutic Chemical code.

Consumption data included year of prescription, patient age group, sex (binary), region of residence, active antibiotic substance, ATC code, number of prescriptions, number of defined daily doses (DDDs), indication, number of treated patients and population size.

Population demographics were analysed between 2012 and 2023 to provide information on 10 years of population size development, the percentage of older inhabitants and the distribution by sex and age.

#### Outcome measures

The main outcome measures of antibiotic consumption were prescriptions per 1,000 people ≥ 70 years (PrEI) and defined daily dose per prescription (DPr). The PrEI allowed for comparison of consumption between populations e.g. age groups and over time. The DPr indicated prescribing behaviour.

#### Classification of prescription indications by infection type

This study included all prescriptions for the four antibiotics presented in Table 1, regardless of indication. The percentage shares of prescriptions specifically indicated for UTIs were calculated to assess the extent to which other infections might have contributed to the prescribing of the four included antibiotics. Prescriptions were divided into: (i) indications for UTI (urinary tract infection, cystitis, pyelonephritis, prevention of urinary tract infection and chronic urinary tract infection), (ii) indications for other types of infections, and (iii) non-informative indications (unknown indication, infection, severe infection and prevention of severe infection). An overview of these indications can be found in Supplementary Table S1.

### National register-based surveillance study of antimicrobial resistance

#### Data source

Antimicrobial resistance data for *E. coli* and *K. pneumoniae* urine isolates obtained from general practice were extracted from the Danish Microbiology Database (MiBa) in September 2024 through the mapped database EpiMiBa [15,17]. The Danish Microbiology Database contains local codes from the Danish Departments of Clinical Microbiology (DCM), whereas EpiMiBa contains nationally mapped codes and can therefore be used for surveillance purposes. Data from EpiMiBa were considered complete from 2018; therefore, this study only covered 2018–2023 [15]. All urine isolates from patients 70 years old and older analysed at any DCM were included.

### Microbiological methods

The susceptibility testing was primarily performed by disc diffusion at the DCMs and results were reported to MiBa [18]. Data from MiBa consisted of interpreted microbiological results based on the susceptible, intermediate and resistant (SIR) system [19].

#### Data handling

Data from MiBa were pre-processed by Data Integration and Analysis at Statens Serum Institut (SSI). The applied case definition was aligned with the European Antimicrobial Resistance Surveillance Network (EARS-Net). Thus, the first isolate from urine from each unique patient and bacterial species per year was included [18]. Susceptibility data from a DCM were excluded if < 75% of a specific pathogen-antibiotic combination had been tested and registered as susceptible (S), intermediate (I) or resistant (R) in MiBa [18].

Data were analysed to assess resistance of *E. coli* and *K. pneumoniae* to the four included antibiotics (Table 1). Percentage resistance rates were calculated based on the number of cases with an R susceptibility result.

### Initiatives to improve diagnosis and treatment of urinary tract infections

A timeline of initiatives to reduce antibiotic consumption in Denmark was constructed for 2005–2020. Initiatives were searched for in the period March to September 2024 and included national campaigns, changes to UTI treatment guidelines and the establishment of relevant councils. National UTI treatment guidelines were found through the Danish Medicines Council and the Danish College of General Practitioners (Dansk Selskab for Almen Medicin). Changes to treatment guidelines were identified through the Danish Health Authority and the Journal of the Danish Medical Association. Information on campaigns to reduce antibiotic use was provided by SSI. Information on national action plans and awareness campaigns was sourced through the Danish information platform ‘Antibiotika eller ej’ (antibiotics or not) [15,20].

### Statistical analysis

All statistical tests were performed by using Excel 2019 version 1808 and a p value below 0.05 was applied to indicate statistical significance.

#### Test for sudden changes in prescription levels

The decline in nitrofurantoin prescriptions around 2017 were assessed by an interrupted time series analysis on the 2012–2023 series. A segmented linear regression was fitted with 2017 as the intervention point, comprising a pre-intervention time trend, a level-change term and a slope-change term, so that the immediate change in prescription level at 2017 and the subsequent change in trend could be estimated separately. The model was estimated by ordinary least squares with Newey–West (heteroskedasticity- and autocorrelation-consistent) standard errors to account for serial dependence between consecutive years. The significance of the level and slope changes was assessed by t-tests on the corresponding regression coefficients, evaluated against a Student's t-distribution with n − k = 8 degrees of freedom.

#### Changes in antimicrobial resistance rates

To detect potential statistical changes in resistance rates of *E. coli* and *K. pneumonia*e to the four included antibiotics (Table 1) over the 6-year period, a Cochran-Armitage test was applied. One-sided tests were applied due to the preliminary observation of declining trends.

## Results

### Demographics

Population demographics showing the development of population size from 2012 to 2023 are presented in Table 2.

**Table 2:** Demographics of the population ≥70 years old, Denmark, 2012 and 2023

Demographics	Population size			Distribution of age and sex within the older population							
Year	Population (N)	Population ≥ 70 years		All 70–79 years		All ≥ 80 years		Female ≥ 70 years		Male ≥ 70 years	
		n	%	n	%	n	%	n	%	n	%
**2012**	5,580,516	628,129	11.3	397,856	63.3	230,363	36.7	360,106	57.3	268,113	42,7
**2023**	5,932,654	887,977	15.0	583,645	65.7	304,332	34.3	486,956	(4.8	401,021	45,2

From 2012 to 2023, the older population increased by 32.7% in Denmark. In 2012, people aged 70 years or above accounted for 11.3% of the total Danish population, rising to 15.0% in 2023. A full overview of demographics from 2012 to 2023 can be found in Supplementary Table S3.

### Antibiotic consumption for urinary tract infection treatment in people ≥ 70 years

#### Classification of prescription indications by infection type

All four antibiotics had high proportions of prescriptions specifically indicated for UTI treatment ranging from 83% to 98%. However, a considerable proportion of trimethoprim prescriptions (17.0%) was not prescribed for UTIs in 2023. Supplementary Table S4 shows that a substantial proportion of the remaining indications for all four antibiotics was non-informative (range: 2─17%), with only a negligible percentage reported for infections other than UTI (<1%). From 2015 to 2023, the indications became increasingly specific, except for trimethoprim, where the proportion of infection-specific indications increased during 2015–2019 but then decreased.

#### Antibiotics for urinary tract infection treatment

**Figure 2:**
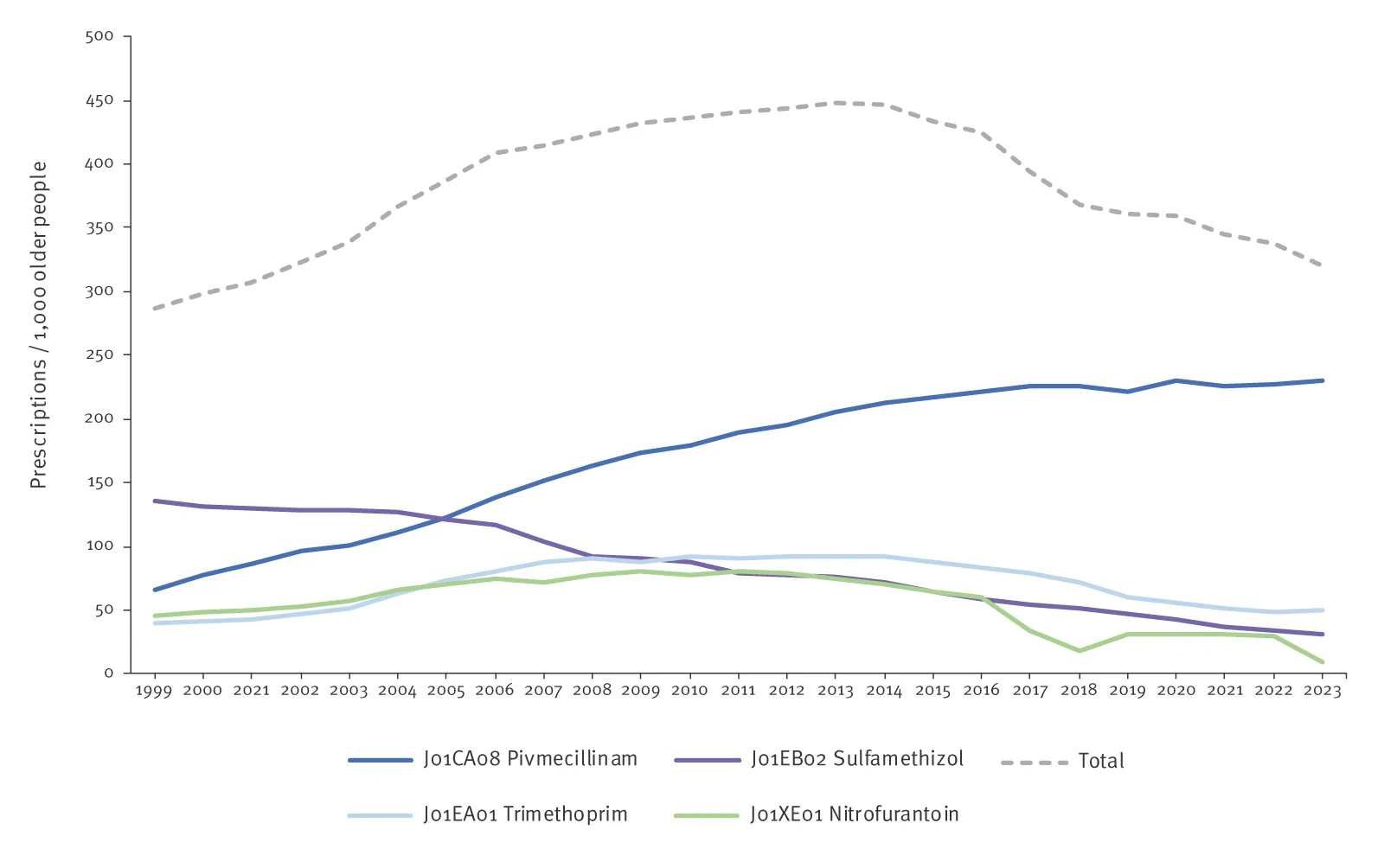
Prescriptions of antibiotics used to treat urinary tract infections in the population ≥ 70 years old in primary healthcare, Denmark, 1999–2023

Consumption of all four antibiotics for UTI treatment increased by 55.8% (287 to 447 PrEI) from 1999 to 2014 and decreased by 28% from 2014 to 2023 (447 to 320 PrEI) (Figure 2).

J01 refers to the Anatomical Therapeutic Chemical code.

When each antibiotic was analysed separately, there was a 3.5-fold increase in pivmecillinam prescribing from 66 PrEI in 1999 to 230 PrEI in 2023 (Figure 2). In 2023, pivmecillinam prescriptions constituted 71.9% of the total consumption for UTI treatments. The highest prescription level of sulfamethizole was observed in 1999 at 136 PrEI and decreased by 77.2% to 31 PrEI in 2023. The number of trimethoprim prescriptions increased by 27.5% from 1999 to 2023 (40 to 51 PrEI). The highest level was observed in 2012 at 91 PrEI, after which it declined by 44.0% by 2023. Nitrofurantoin prescription levels ranged from 46 to 81 PrEI from 1999 to 2016. After 2016 a marked decrease was observed, from 60 PrEI in 2016 to 34 PrEI in 2017 and further to 18 PrEI in 2018. The interrupted time series analysis revealed a significant immediate drop in prescription level in 2017 (p = 1.98×10^−3^), while the change in trends after 2017 was not significant (p = 0.15).

Analysis of the consumption of the four antibiotics in older people by age group showed that consumption among those aged 80 years or older (488 PrEI) was ca 2-times higher than among the 70–79-year-olds (232 PrEI) in 2023. The number of prescriptions for the 70–79-year-olds and the 80-year-olds and older decreased by 20.9% and 30.2%, respectively, from 2012 to 2023. Supplementary Figure S1 shows the decreasing trends in antimicrobial consumption by the two age subgroups of older people.

### Antibiotic resistance in *Escherichia coli* and *Klebsiella pneumoniae*

**Figure 3:**
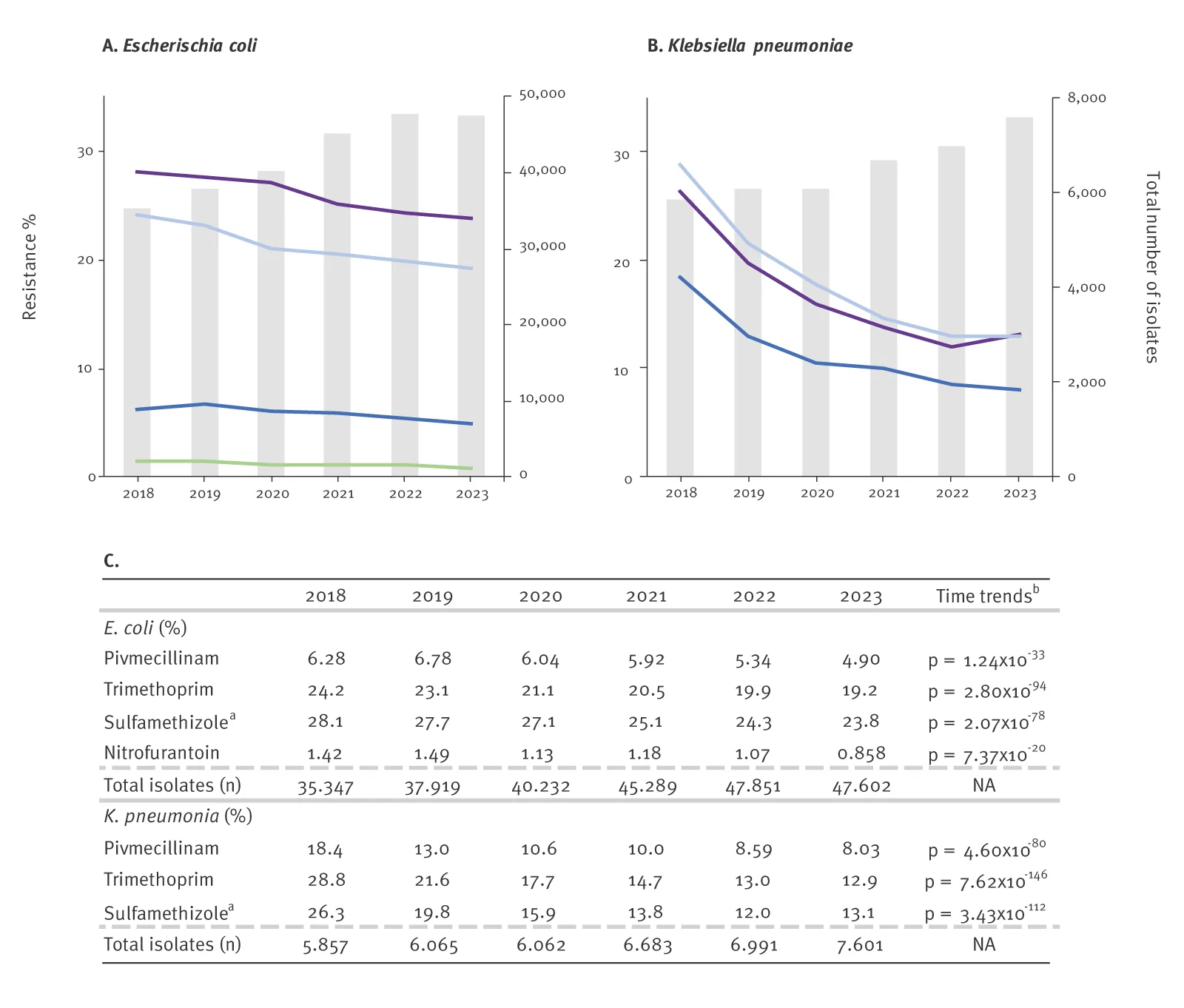
Annual number of urine isolates and proportion of resistance in *Escherichia coli* and *Klebsiella pneumoniae* in patients 70 years and older in primary healthcare, Denmark, 2018–2023

Resistance data on *E. coli* and *K. pneumoniae* to pivmecillinam, sulfamethizole, trimethoprim and nitrofurantoin between 2018 and 2023 in Denmark are shown in Figure 3. There is no clinical breakpoint for nitrofurantoin in the European Committee on Antimicrobial Susceptibility Testing (EUCAST) guidelines for *K. pneumoniae* and the tested fraction did not reach > 75% [21]. Thus, resistance for this antibiotic-pathogen combination is not presented.

NA: not applicable.

^a^ No standard European Committee on Antimicrobial Susceptibility Testing clinical breakpoints for categorizing microorganisms as susceptible (S-I-R) to sulfamethizole.

^b^ Cochran-Armitage trend test.

Resistance to trimethoprim and sulfamethizole was notably high in both species. A decrease in resistance rates for all four antibiotics was observed for both bacteria from 2018 to 2023. The Cochran-Armitage test revealed a statistically significant downward trend in resistance rates for all four antibiotics from 2018 to 2023. Simultaneously, the total number of analysed *E. coli* and *K. pneumoniae* isolates increased by 34.7% and 29.8%, respectively.

### Initiatives to improve diagnosis and treatment of urinary tract infections

**Figure 4:**
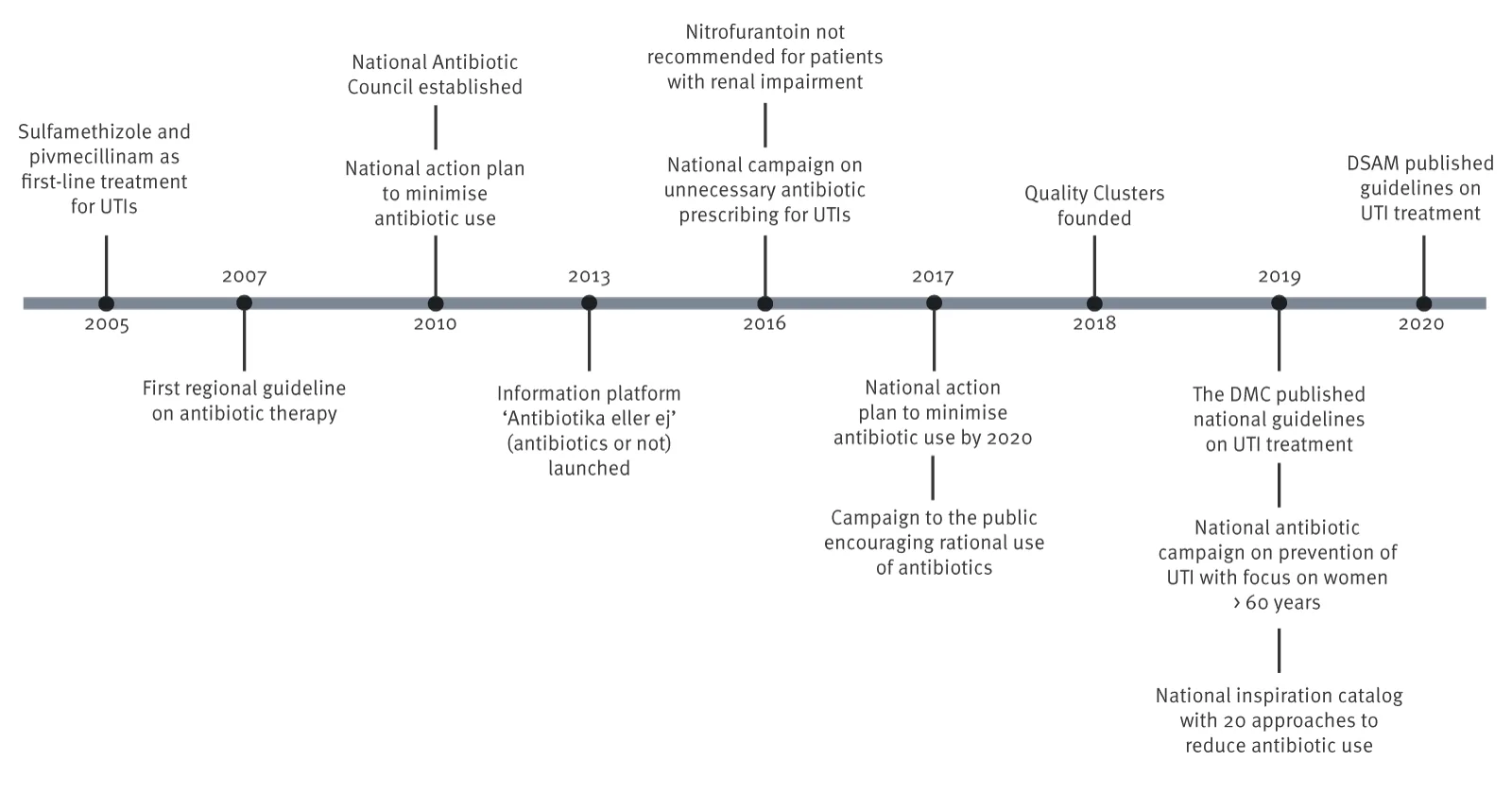
Timeline of national initiatives and guidelines on antibiotic treatment of urinary tract infections, Denmark, 2005–2020

DMC: Danish Medicinal Council; DSAM: Danish College of General Practitioners; UTI: urinary tract infection.

Figure 4 presents a timeline of national initiatives to improve antibiotic treatment of UTIs from 2005 to 2020. The COVID-19 pandemic years (2020–2022) halted the initiatives due to increased focus on pandemic management. However, the generally increased focus on infectious diseases post pandemic was beneficial and led to several initiatives that were still under negotiation at the time of the research and therefore not included [15].

Quality Cluster is a forum for general practitioners based on geographic location with the purpose of improving quality in general practice.

## Discussion

This study investigated antibiotic consumption for UTIs among adults aged ≥70 years in Danish primary healthcare (1999–2023) and AMR in urinary *E. coli* and *K. pneumoniae* isolates (2018–2023), contextualised by a timeline of key antimicrobial stewardship and UTI-related initiatives. The results showed a peak in antibiotic prescriptions for UTIs (447 PrEI) in 2014 followed by a 28.4% decrease by 2023. Simultaneously, the percentage of AMR in urinary *E. coli* and *K. pneumoniae* isolates decreased from 2018 to 2023. The decreases may reflect the effectiveness of initiatives since 2013 targeting the public and healthcare professionals to reduce antibiotic consumption in Denmark [5,11,14,20,22-24]. Around 2005, the increased prevalence of sulfamethizole-resistant *E. coli* resulted in microbiologists recommending to switch from sulfamethizole to pivmecillinam as first-line treatment in general practice. This resulted in increased consumption of pivmecillinam, despite the overall decrease in total prescription numbers for UTIs [25,26]. The increasing consumption of pivmecillinam was followed by a decrease in the proportion of pivmecillinam-resistant isolates. This is counterintuitive, as higher consumption often leads to increased AMR [27]. *E. coli* develops resistance to pivmecillinam in vitro, but resistance remains low in vivo [28], potentially explained by the fitness cost of the mecillinam-resistant mutant. The resistant mutant has a low growth rate and therefore a reduced ability to survive in the bladder [28]. The low rate of resistance indicates that pivmecillinam remains a rational first-line treatment for UTIs. Pivmecillinam constituted the majority of prescriptions in 2023 (71.9%), indicating compliance with current guidelines which are appended in Supplementary Table S2.

Two declines were observed in nitrofurantoin consumption between 1999 and 2023. In 2016, a new recommendation advised against the use of nitrofurantoin in older patients due to increased risk of lung fibrosis in cases of impaired kidney function [29]. This recommendation resulted in decreased nitrofurantoin consumption until 2018. In 2019, consumption increased again, probably due to guidelines not being followed to the same extent as when they are newly implemented. A medicine shortage affected nitrofurantoin in 2023 and was likely an additional cause of the notable decline observed in that year. The shortage could explain the increase in DDD per prescription in 2023 for nitrofurantoin, where general practitioners or pharmacies may have substituted with the available larger packages [30].

Despite the trends towards a decrease in resistance, *E. coli* and *K. pneumoniae* exhibited high resistance rates to trimethoprim and sulfamethizole. The high sulfamethizole resistance is expected because of previous widespread use. However, trimethoprim has not previously been used to the same extent. The high trimethoprim resistance is likely explained by cross-resistance. The mechanisms of action of trimethoprim and sulfamethizole are similar, which enables bacteria to develop resistance towards both drugs simultaneously [31,32]. Nonetheless, resistance rates for both drugs have decreased in line with the decrease in consumption from 2018 to 2023.

Other European surveillance programs do not present antimicrobial consumption and resistance data specifically for the population ≥70 years old. However, in Norway, total consumption of pivmecillinam independent of age has decreased slowly since 2014 and trimethoprim since 2012, whereas nitrofurantoin has fluctuated over the years [33]. Resistance levels are comparable with the Danish figures. However, the change in the number of urinary isolates in primary healthcare was not presented, which may influence the expected burden of UTIs in the near future. In the Netherlands, surveillance data from LTFCs showed overall decreased consumption of antimicrobials since 2018, but with increased consumption in 2023 compared with the previous year. The commonly used penicillin combinations (amoxicillin with clavulanic acid), fluoroquinolones and nitrofurantoin reflect that UTIs are the most common infection at LTCFs in the Netherlands. Nitrofurantoin is first-line treatment for an uncomplicated UTI which shows a low level of resistance in *E. coli* (3%) [34].

Previous studies have shown that the people ≥80 years old and residents at LTCFs have the highest antibiotic consumption [3,35], possibly explained by age-related physiological changes, polypharmacy and comorbidities. Arnold et al. showed that interventions targeting UTIs generally succeed in reducing antibiotic prescription levels in LTCFs [36]. In line with this, the most notable declines in PrEI were observed for those 80 years and older, indicating that increased national and regional focus on rational pharmacotherapy and hygiene challenges have a positive impact on antimicrobial consumption [14].

It is well-described that diagnostic processes in LTCFs can be challenging [3]. The prevalence of ABU is higher among people in LTCFs due to certain medical conditions and is easily misdiagnosed as a UTI [35,37]. To promote prudent antibiotic use and prevent the treatment of ABU, the national guidelines specify that antibiotics should not be prescribed without urine examination and urine examination should not be considered without typical UTI symptoms [5,11]. The higher antibiotic consumption in this population may be explained by a higher disease burden associated with multimorbidity and polypharmacy [12]. However, Azaizi et al. showed that the high antibiotic prescription levels among the older population in Danish LTCFs were not solely explained by differences in age, sex or comorbidities [3]. This indicates that additional factors are driving the difference and potential overuse. These may include the complexity of diagnosing in LTCFs and different approaches of antibiotic prescribing among general practitioners [3].

Sommer-Larsen et al. showed that less than half of the patients in LTCFs diagnosed with UTIs had symptoms and that most patients without symptoms had a urine culture performed nonetheless [38]. This may lead to inappropriate antibiotic prescribing. Weatherall et al. demonstrated that integrating nursing home physicians can improve patient care and health outcomes [39]. An estimate of 70–75% of all LTCFs in Demark had a nursing home physician in 2022 [40], leaving the diagnosis and treatment capacity of the remaining LTCFs dependent on the knowledge and experience of the LTCF staff [41].

The increase in number of urinary isolates from 2018 to 2023 indicates that, despite a decrease in resistance, the total number of infections may be increasing. Over time, the increasing number of isolates may strain healthcare resources because of quantitively more infections to treat and thus more infections with resistant bacteria [1]. The results of this study also show demographic changes, with an increase in the older population from 2018 to 2023 (12.1%), which may have led to a higher number of urine samples being tested as more people are susceptible to infection [42]. The results therefore suggest that although AMR levels have decreased, there may still be challenges in the future due to a rise in the number of infections. It should be noted, however, that the rise in the number of isolates could also be influenced by changes in the diagnostic process resulting in increased testing at DCMs instead of local point-of-care testing.

A major strength of this study is the national antibiotic prescription dataset because of its validity and completeness [43]. Additionally, the infection-specific indications revealed that pivmecillinam, sulfamethizole and nitrofurantoin were almost exclusively used in UTI treatment and most trimethoprim indications were also for UTI treatment. However, only redeemed prescriptions were included; unredeemed prescriptions therefore do not affect the results of this study.

The study is limited to consumption of the four selected antibiotics in general practice and does not offer a complete overview of antibiotic consumption for UTI treatment in Denmark. Data are limited to prescription sales to individuals with a Danish civil registration number and do not include sales to clinics (3–4%) [44]. Additionally, antibiotic consumption is measured in prescriptions, which may not reflect the actual consumption by patients.

Susceptibility testing from the DCMs provides valuable data on AMR patterns in *E. coli* and *K. pneumoniae*. However, the data do not provide a complete overview of AMR and infection rates as only the first isolate from urine per patient per year was included. Additionally, susceptibility testing performed locally in general practice was not included. Resistance data only extended back to 2018 as the EpiMiBa register was considered complete from this year.

The study period coincides with the COVID-19 pandemic in 2020–2022. However, since the pandemic is not the main focus of the study, we have not included it in our methods or discussion. Nevertheless, it is possible that the observed trends in this study could have been affected by pandemic-related infection prevention and control measures, reduced foreign travel, changes in healthcare-seeking behaviour and restricted access to healthcare providers.

## Conclusion

This study has identified patterns in antibiotic consumption and resistance within the ≥70 years old Danish population. The results demonstrate a decrease in antibiotic prescription numbers for UTI treatment in Danish primary healthcare since 2014. Simultaneously, the percentage of AMR in *E. coli* and *K. pneumoniae* has decreased. These results could reflect the impact of antibiotic stewardship initiatives implemented to improve diagnosis and treatment of UTIs.

This study also demonstrates a rise in the number of urine isolates, which may indicate increasing infection rates or structural changes in the diagnostic process. Additionally, the older population increased over the study period, resulting in a higher number of individuals at risk of UTI. This indicates that while AMR in UTIs may not pose an acute threat at present, it could strain healthcare resources in the near future.

## Data Availability

Aggregated data are available through request to DANMAP (www.danmap.org). All data presented in the manuscript or supplementary material are available from the corresponding author upon reasonable request.

## Supplementary Data

202000 Supplementary Material
