# Supplementary material for: Antibiotic treatment and antimicrobial resistance in urinary tract infections among the older population in Denmark, 1999 to 2023
Source: Euro Surveill. 2026 Aug 20;31(33):2500801. doi: 10.2807/1560-7917.ES.2026.31.33.2500801 (PMC13498900; doi:10.2807/1560-7917.ES.2026.31.33.2500801)
Supplement: Supplementary file 1 [file 25-00801_ATTAUABI_Supplement.pdf]

Supplementary material

This supplementary material is hosted by *Eurosurveillance* as supporting information alongside the article 'Antibiotic treatment and antimicrobial resistance in urinary tract infections among the older population in Denmark, 1999 to 2023', on behalf of the authors, who remain responsible for the accuracy and appropriateness of the content. The same standards for ethics, copyright, attributions and permissions as for the article apply. Supplements are not edited by *Eurosurveillance* and the journal is not responsible for the maintenance of any links or email addresses provided therein.

Supplementary Table S1. Overview of grouped urinary tract infection indications as registered in Danish Register of Pharmaceutical Sales.

Indication group	Indication Code	Description
Urinary tract infection		
	0000103	Against cystitis
	0000104	Against urinary tract infection
	0000448	Against pyelonephritis
Prevention of urinary tract infection		
	0000126	Prevention of urinary tract infection
Chronic urinary tract infection		
	0000264	Against chronic urinary tract infection
Infection		
	0000084	Against infection
	0000312/0000062	Against inflammation
	0000805	Against inflammation and infection
Severe infection		
	0000124	Against severe infection
	0000482	Against cerebral abscess
	0000426	Against meningitis
	0000741	Against anthrax
	0000652	Treatment of anthrax
	0000479	Against sepsis
Prevention of severe infection		
	0000486	Prevention of severe infection
	0000296	Prevention of meningitis
	0000132	Prevention against meningitis

Supplementary Table S2. Regional treatment guidelines for urinary tract infections in primary health care, Denmark.

	Capital Region of Denmark	Central Region of Denmark	North Denmark Region	Region Zealand	Region of Southern Denmark
**Uncomplicated urinary tract infection**	Pivmecillinam 400 mg 3 times daily for 3 days.<br><br>Penicillin allergy:<br>Nitrofurantoin 50-100 mg 4 times daily for 3 days or trimethoprim 200 mg 2 times daily for 3 days.<br><br>Sulfamethizole can be used if susceptibility testing confirms its effectiveness[1]	Pivmecillinam 400 mg 3 times daily for 3 days.<br><br>Penicillin allergy:<br>Trimethoprim 200 mg 2 times daily for 3 days.<br><br>Other: Nitrofurantoin[1].	Pivmecillinam 400 mg, 3 times daily, for 3 days.<br><br>Penicillin allergy:<br>Nitrofurantoin (GFR >45 mL/min) or trimethoprim[1]	Pivmecillinam 400 mg, 3 times daily, for 3 days.<br><br>Penicillin allergy:<br>Susceptibility testing.<br><br>Nitrofurantoin 50 mg 4 times daily for 3 days or trimethoprim 200 mg 2 times daily for 3 days.<br><br>Sulfamethizole can be used if susceptibility testing confirms its effectiveness[1]	Pivmecillinam[2]<br><br>Penicillin allergy:<br>Nitrofurantoin for 3 days[2]
**Complicated urinary tract infections**	Pivmecillinam 400 mg 3 times daily for 5 days.<br><br>Penicillin allergy:<br>Nitrofurantoin 50-100 mg 4 times daily for 5 days (GFR >45 mL/min)	Pivmecillinam 400 mg 3 times daily for 5 days.<br><br>Penicillin allergy:<br>Nitrofurantoin 50-100 mg 4 times daily for 5 days (GFR >45 mL/min)<br><br>Ciprofloxacin 500 mg 2 times daily if GFR < 45mL/min	Pivmecillinam 400 mg, 3 times daily, for 5 days.<br><br>Penicillin allergy:<br>Nitrofurantoin (GFR >45 mL/min)[1]<br><br>Ciprofloxacin can be used if susceptibility testing confirms its effectiveness[1]	Pivmecillinam 400 mg, 3 times daily, for 5 days.<br><br>Penicillin allergy:<br>Susceptibility testing.<br><br>Nitrofurantoin 50 mg 4 times daily for 5 days (GFR >45 ml/min)<br><br>Ciprofloxacin 500 mg 2 times daily if GFR < 45ml/min	Pivmecillinam[2]<br><br>Penicillin allergy:<br>Nitrofurantoin for 5 days[2]
**Acute Pyelonephritis**	Pivmecillinam 400 mg 3 times daily for 7 days.<br><br>Penicillin allergy:<br>Ciprofloxacin 500 mg 2 times daily for 7 days.	Pivmecillinam 400 mg 3 times daily for 7 days.<br><br>Penicillin allergy:<br>Ciprofloxacin 500 mg 2 times daily for 7 days.	N/A[3]	Pivmecillinam 400 mg 4 times daily for 7-10 days.<br><br>Penicillin allergy: Ciprofloxacin 500 mg 2 times daily for 7 days.	Pivmecillinam[2]<br><br>Penicillin allergy:<br>Ciprofloxacin[2]
**Prophylaxis**	Long-term prophylaxis with antibiotics generally not recommended. May be considered for frequent, significantly bothersome recurrent bacterial cystitis: Pivmecillinam 200 mg at night or trimethoprim 100 mg at night every 3 months to prevent resistance development. A break is recommended after 6 months.	Long-term prophylaxis with antibiotics not recommended.	N/A[3]	Can be treated with trimethoprim or nitrofurantoin. The antibiotics should be alternated every 4-6 weeks to avoid resistance. Nitrofurantoin should not be used in case of reduced kidney function.	N/A[2]

**References**	Urinveje (Antibiotikavejledning for primærsektoren - Region Hovedstaden) [Internet]. Copenhagen: Sundhed.dk. [Cited 2024 Aug 15]. Available from: https://www.sundhed.dk/sundhedsfaglig/information-til-praksis/hovedstaden/almen-praksis/patientbehandling/laegemidler/antibiotikavejledning/a07-urinveje/	Infektioner i almen praksis [Internet]. Region Midtjylland: Midtkraft; Version 3, 2024. [Cited 2024 Aug 27]. Available from: https://midtkraft.dk/wp-content/uploads/2024/04/w_8480_Infektioner-i-almen-praksis_pjece_A5.pdf	Infektioner (basislisten - lægemiddelanbefalinger i Region Nordjylland) [Internet]. Aalborg: Sundhed.dk;2023. [Updated 2023 Feb 24, cited 2024 Jun 27] Available from: https://www.sundhed.dk/sundhedsfaglig/information-til-praksis/nordjylland/almen-praksis/patientbehandling/laegemidler/basislisten-nordjylland/o-infektioner/	Infektioner i almen praksis [Internet]. Sorø: Region Sjælland; Version 6, 3. udgave 2024. [Cited 2019 Aug 12]. Available from: https://www.sundhed.dk/content/cms/93/134793_infektioner-i-almen-praksis-2024-version-6-udgave-3.pdf<br><br>Infektioner (A) (basislisten - lægemiddelanbefalinger i Region Sjælland) [Internet]. Sorø: Sundhed.dk; 2024. [Updated 2024 Jun 3, cited 2024 Sep 19] Available from: https://www.sundhed.dk/sundhedsfaglig/information-til-praksis/sjaelland/almen-praksis/patientbehandling/laegemidler/basislisten-sjaelland/basislisten-efter-i-n/infektioner/	Infektioner (basislisten - lægemiddelanbefalinger i Region Syddanmark) [Internet]. Vejle: Sundhed.dk; 2024. [Updated 2024 Jul 9, cited 2024 Sep 19] Available from: https://www.sundhed.dk/sundhedsfaglig/information-til-praksis/syddanmark/almen-praksis/patientbehandling/laegemidler/basislisten/basislisten-indikationer-syddanmark/infektioner/<br><br>Urinvejsinfektioner i almen praksis [Internet]. Copenhagen: DSAM; 2020. [Updated 2020 Sep cited 2024 Sep 19]. Available from: https://www.dsam.dk/vejledninger/urinvejsinfektioner/urinvejsinfektioner-i-almen-praksis#behandling

[1]Lack of information on drug strength or duration of treatment. [2]The Southern Region of Denmark's essential medicine list for UTI treatment [Basislisten] did not mention duration or strength however the region refers to DSAM's treatment guidelines. [3]Regional guidelines did not mention treatment of this indication. Abbreviations: GFR: Globular filtration rate.

Supplementary Table S3. Population demographics, Denmark, 2012-2023.

	Population data		Distribution of sex and age within the elderly population		
**Year**	**Population (no)**	**Elderly Population (%)**	**70-79 years (%)**	**≥ 80 years (%)**	**Female (%)**
**2012**	5,580,516	628,129 (11.3)	63.3	36.7	57.3
**2013**	5,602,628	647,766 (11.6)	64.1	35.9	56.9
**2014**	5,627,235	670,442 (11.9)	65.0	35.0	56.6
**2015**	5,659,715	699,249 (12.4)	65.7	34.2	56.2
**2016**	5,707,251	730,074 (12.8)	66.6	33.4	55.9
**2017**	5,748,769	762,876 (13.3)	67.3	32.7	55.5
**2018**	5,781,190	792,435 (13.7)	67.6	32.4	55.3
**2019**	5,806,081	815,804 (14.1)	67.7	32.3	55.1
**2020**	5,822,763	836,716 (14.4)	67.5	32.5	55.0
**2021**	5,840,045	856,803 (14.7)	67.1	32.9	54.9
**2022**	5,873,420	872,524 (14.9)	66.6	33.4	54.9
**2023**	5,932,654	887,977 (15.0)	65.7	34.3	54.8

Supplementary Table S4. Specificity of indications of pivmecillinam, trimethoprim, sulfamethizole and nitrofurantoin, Denmark, 2015-2023.

	**2015**	**2016**	**2017**	**2018**	**2019**	**2020**	**2021**	**2022**	**2023**
**Pivmecillinam**									
UTI (%)	86.0	88.0	92.6	94.7	94.9	95.5	96.6	97.4	97.7
Other (%)	0.2	0.2	0.0	0.2	0.1	0.1	0.1	0.2	0.1
Unknown/infection (%)	13.8	11.8	7.4	5.2	5	4.4	3.3	2.4	2.2
**Sulfamethizole**									
UTI (%)	95.7	92.4	95.4	97.2	97.7	97.7	97.7	97.7	97.5
Other (%)	0.1	0.1	0.0	0.0	0.0	0.0	0.0	0.0	0.1
Unknown/infection (%)	4.2	7.5	4.6	2.7	2.3	2.3	2.2	2.3	2.4
**Trimethoprim**									
UTI (%)	83.6	84.3	87.1	89.4	90.4	85.6	82.9	82.8	83.0
Other (%)	0.0	0.0	0.0	0.0	0.0	0.0	0.0	0.0	0.0
Unknown/infection (%)	16.4	15.7	12.9	10.6	9.6	14.4	17.1	17.2	17.0
**Nitrofurantoin**									
UTI (%)	87.2	86.9	88.3	92.7	94.8	93.9	93.5	94.5	92.4
Other (%)	0.0	0.0	0.0	0.0	0.0	0.0	0.0	0.0	0.0
Unknown/infection (%)	12.8	13.1	11.7	7.3	5.2	6.1	6.5	5.5	7.6

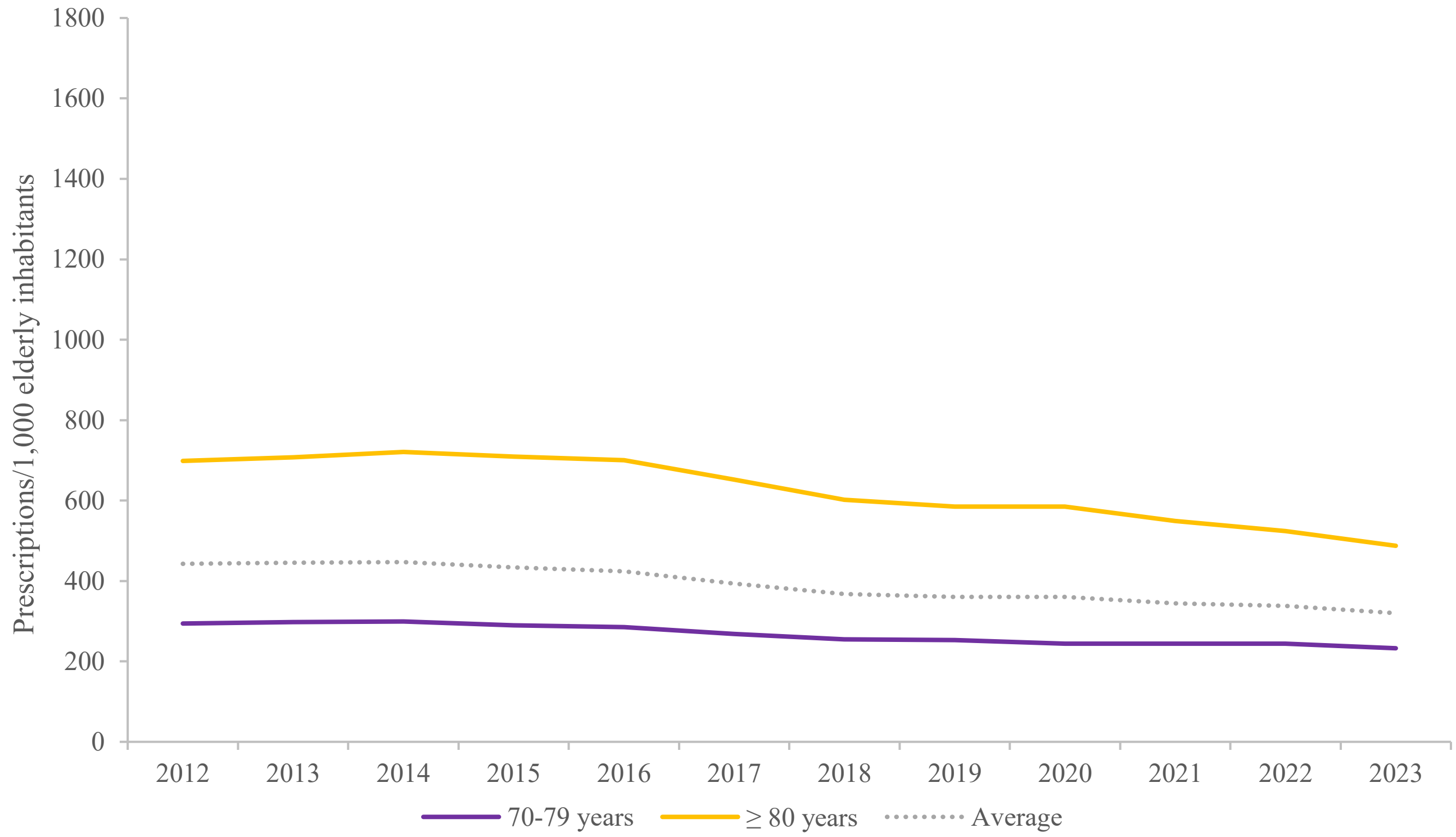

Supplementary Figure S1. Consumption of antibiotics used to treat urinary tract infections (pivmecillinam, trimethoprim, sulfamethizole, and nitrofurantoin) in elderly divided in age groups (70-79 years and ≥ 80 years), Denmark, 2012-2023.
